# Biofilm addition improves sand strength over a wide range of saturations

**DOI:** 10.1016/j.bioflm.2021.100050

**Published:** 2021-06-10

**Authors:** Ahmad Faysal Shariq, Haluk Beyenal, Idil Deniz Akin

**Affiliations:** a Washington State University, Department of Civil and Environmental Engineering, Pullman, WA, 99164, USA; b Washington State University, Gene and Linda Voiland School of Chemical Engineering and Bioengineering, Pullman, WA, 99164, USA; c Colf Distinguished Professor in Geotechnical Engineering, Washington State University, Department of Civil and Environmental Engineering, Pullman, WA, 99164, USA

**Keywords:** Suction stress, Biofilm, Sand, Adsorption, Water retention, Granule, EPS

## Abstract

Bio-mediated ground improvement is an attractive alternative to traditional admixtures for strength improvement of shallow surfaces because it is environmentally friendly. Since biofilms contain extracellular polymeric substances (EPS), they can be considered as an alternative to current technologies to improve soil strength. EPS containing biofilms are porous materials with charged surfaces, and therefore adsorption and capillary condensation can result in water retention. Currently, most of the literature work only tested soil improvement under the dry condition. Therefore, the influence of water retention by biofilms on the strength of improved soil remains unclear. Our goal is to evaluate the strength of a biofilm-enhanced sand over a wide range of saturations and explain the trends through the suction stress characteristic curve (SSCC), which quantifies interparticle adsorptive, capillary, and cementation forces as a function of saturation. We used homogenized anaerobic granule biofilms from an existing upflow anaerobic sludge blanket reactor and mixed it with sand to test the strength of a poorly-graded sand in a wide range of saturations, *S* (between 0.02 *S* and 0.74 *S*). We found that biofilm treatment of sand increases soil strength through cementation over a wide range of saturations and through adsorptive forces among sand, biofilm surfaces, and water molecules at low saturations (*S* < ~0.3). Our results suggested that homogenized biofilms mixed with sand can be used to improve the strength of sand over a wide range of saturations.

## Introduction

Environmental concerns regarding the additives traditionally used in chemical soil modification techniques have resulted in the search for more environmentally friendly bio-mediation techniques (e.g., Refs. [1,2]). Most of the bio-mediated soil improvement research so far has focused on microbially induced calcite precipitation (MICP) in coarse-grained soils. In this process, calcium carbonate is precipitated at particle contacts, typically through urea hydrolysis or denitrification (e.g., Refs. [[3], [4], [5]]). Commercially-available biopolymers such as xanthan gum and gellan gum are also tested for soil strength improvement and shown to be effective (e.g., Ref. [6], [7]). For example [8], found strength improvement with xanthan gum is comparable to that of cement even at low concentrations, as low as 1%. Since biofilms contain extracellular polymeric substances (EPS), they are also considered an alternative to current technologies to improve soil strength.

The research efforts on soil improvement using biofilms have primarily focused on hydraulic conductivity reduction (e.g., Refs. [[9], [10], [11], [12], [13]]). Laboratory testing of the effects of biofilm growth on soil behavior requires a more intensive effort because of the complications associated with monitoring of biofilm growth. In reactors, biofilms are typically grown by circulating a nutrient solution in soil columns mixed with selected microbial communities (e.g., Refs. [9,13,14]). Clogging of tubing due to uncontrolled biofilm growth or to the formation of preferential flow paths in soil columns can require extra precautions during lab testing. The literature review given on the Supplementary Information (SI) Table 1 revealed that 1) most of the literature studies used pure cultures and considered saturated or dry conditions for biofilm growth and strength testing, which may not be relevant to field applications, 2) most of these works grew the biofilm in the soil first then tested soil improvement. To improve soil strength, it is a common practice for civil engineers to mix the soil with an admixture such as a biopolymer.

Improved strength due to an admixture is typically evaluated using remolded specimens compacted at a fixed water content and cured to the dry condition (e.g., Refs. [[15], [16], [17]]). While this approach gives comparisons between treated and untreated soils and may be effective for deep ground improvement, shallow surfaces' field strength cannot be represented accurately. The majority of soil dealt with in shallow geotechnical engineering applications is in the unsaturated zone (i.e., the zone in which air and water coexist in soil pores and pore water pressures are negative), where saturation is dynamic. A change in saturation (or suction) results in a change in soil strength. In this case, the strength of soils is quantified through effective stress (i.e., stress transmitted from one soil grain to another), and the effect of saturation on effective stress can be quantified by the term suction stress, which represents interparticle adsorptive and capillary forces, cementation, and pore water pressure, and is a function of saturation called the suction stress characteristic curve, SSCC (e.g., Ref. [18]).

At low saturations, the forces that control suction stress are adsorptive, and at high saturations, capillary forces are dominant (i.e., [[19], [20], [21]]). Recent studies have shown that the adsorptive and capillary components of suction stress change in a non-monotonic way as saturation increases from 0 to 1 [21,22]. The adsorptive forces are maximal when the soil is dry and decrease exponentially as saturation increases. The capillary forces are zero in the dry and saturated states and reach a maximum at some saturation in between. For clean sand, the adsorptive forces are negligible, and if there is no cementation, suction stress is controlled purely by capillary forces. For materials with charged surfaces, such as clays and polymers, adsorptive forces control suction stress at low saturations. The cementation component of suction stress is considered to be unaffected by saturation [18]. Water vapor sorption isotherms give information on the interaction between water molecules and material surfaces and are unique for each soil (e.g., Ref. [23]). Therefore, they can be used to evaluate the adsorptive forces between material surfaces and water molecules.

The current literature lacks an understanding of how biofilms improve soil strength under a range of saturations. Our goal is to evaluate the reuse of a waste granule biofilm as an alternative means of improving the strength of shallow surface soil. A poorly-graded sand was used in experiments because it is inert (i.e., no physicochemical interaction with the biofilms) and easy to standardize. Anaerobic granules rich in EPS were obtained from an existing upflow anaerobic sludge blanket (UASB) reactor. First, the granule biofilms were homogenized in such a way that the cells and EPS were separated from the granules, then mixed with sand. The sand-biofilm mixture was compacted and allowed to equilibrate to various saturations. The SSCC quantified using unconfined compression test and direct shear test results was used to evaluate the improvement in soil strength of a poorly graded fine sand in a wide range of saturation (i.e., between 0 and 0.74 saturation). The SSCC of untreated sand was also quantified and the SSCC of the biofilm-enhanced sand was compared with that of untreated sand to investigate the mechanisms that result in improved strength. Water vapor sorption isotherms were used to evaluate the adsorptive forces between water and biofilm-enhanced sand. The nonlinear trend in the SSCC of biofilm-enhanced sand was explained using the water uptake mechanism (adsorption and capillary condensation) of biofilms.

## Methods and materials

### Sand

The soil is a poorly graded white sand classified as SP (poorly-graded sand) according to the Unified Soil Classification System. The specific gravity was measured according to ASTM D854 as 2.61. The maximum and minimum void ratios of the sand were measured according to ASTM D4254 as 0.91 and 0.65.

### Biofilms

**Fig. 1:**
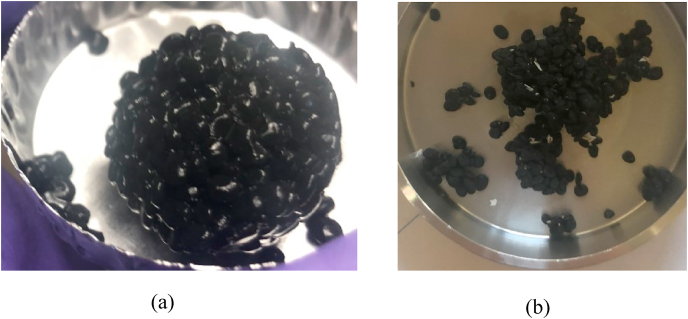
Granules: (a) saturated, ready to be mixed with soil after homogenization, and (b) at 3% relative humidity (RH).

The granule biofilms were obtained from an existing UASB reactor (Penford Food Ingredients Co., Richland, WA). In this reactor, the granule biofilms were grown anaerobically on starch industry wastewater [24]. The major groups of microorganisms in this ecosystem are fermentative bacteria, acidogenic bacteria, acetogenic bacteria, hydrogenophilic and hydrogenotrophic species, and aceticlastic and acetotrophic species [24]. We should note that these granules were enriched using potato processing waste. Using granules from a UASB reactor fed with different waste could potentially generate different biofilm structures and microbial communities. The diameters of the individual granules were between 1 mm and 2 mm. When saturated, a granule is soft and flexible (Fig. 1a). Upon drying, the granule shrinks (~0.5-mm diameter) and turns into a rigid solid (Fig. 1b). The dry density and water content of biofilm granules were 28.7 g/L and 97.7%, respectively. We used standard methods to determine the dry density and water content of the granules [25]. Since we homogenized the granules and exposed the microbial communities to oxygen, microbial growth is negligible.

### Specimen preparation

The biofilm granules were taken from the UASB reactor in Penford and then stored in the laboratory at 4 °C. Before preparing each biofilm sample, the suspension was mixed well. The biofilm granules were separated from the excess liquid by centrifuging the suspension at 10,000 rpm for 3 min and discarding the excess liquid. A 1% yeast extract solution (Bacto™, ThermoFisher catalog #212750) was then mixed with the biofilm granules using a vortex mixer, and the granules were homogenized for 1 min to ensure all the cells were dispersed. Dry sand was then added and mixed thoroughly for 5 min. To ensure a constant void ratio among all specimens, 91.6 g of dry sand was mixed with 22.9 g of homogenized biofilm granules and 13 ml of the 1% yeast solution. Void ratio is defined as the ratio of pore volume between the sand particles to the volume of the sand particles. The biofilm pores are not included in the void ratio calculations. Cylindrical specimens (3.45-cm diameter, 9.19-cm height) were compacted in three layers by applying dynamic load with a 9.07 kg tamper. The load was applied 25 times for each layer.

The specimens were extracted immediately after compaction and were left at room temperature for various durations from 4 h to 120 h to air-dry to different saturations. Water content decreased exponentially with air-drying time as shown in Fig. SI-1. The final degree of saturation for each specimen was calculated after the unconfined compression test using the water content measured at the mid-point of the specimen. The diameter and height of the specimens were measured after drying to calculate the final void ratio and saturation of the specimens. The specimens were dried to a wide range of saturations between 0.74 and 0.02. Duplicates and triplicates were prepared at a number of saturations. After air-drying, specimens were kept in sealed bags for 48 h to promote homogeneous moisture distribution along the specimens, and then tested for unconfined compressive strength.

### Soil water retention curve and SSCC of untreated sand

The soil water retention curve (SWRC) of the sand was measured with the transient release and imbibition method (TRIM) [26] to quantify the SSCC of the untreated sand. The TRIM uses the axis translation method to measure the transient outflow response of soil after it is exposed to a large change in suction. Dry sand was compacted to a 0.85 void ratio inside the TRIM flow cell (6.18-cm diameter, 2.54-cm height) and back-saturated. A drying test was performed by applying a sudden increase in suction, first to 3 kPa and then to 200 kPa. The outflow due to the increase in suction was measured over time using an electronic balance. The transient outflow response was used as an input in a numerical model that solves Richard's equation. The solution of inverse modeling gave the SWRC.

The SSCC of the untreated sand was calculated from the SWRC to compare with the SSCC of the biofilm-treated sand according to the commonly used equation proposed by Ref. [27]:

(1) $$\sigma^{s}=S_{e}\psi$$

where *ψ* is the matric suction and *S*_*e*_ is the effective saturation calculated using the [28] SWRC model:

(2) $$S_{e}=\frac{S-S_{r}}{1-S_{r}}={\left[\frac{1}{1+{\left(\alpha \psi \right)}^{n}}\right]}^{\left(1-\frac{1}{n}\right)}$$

where *S*_*r*_ is the residual saturation and *α, n*, and *m* are fitting parameters to the SWRC. Parameter *α* (kPa^−1^) is related to the inverse of the air entry pressure (AEP) and *n* is related to pore size distribution [29]. Eqn. (1) has been shown to be in good agreement with experimental SSCC for sands [21,27], and was selected to be used in this study because of its simple form.

Although Eqn. (1) can be used to convert between the SWRC and SSCC for pure sand, it cannot be used for biofilm-treated sand. Eqn. (1) represents the contribution of capillary forces to suction stress and is valid for materials which only have capillarity as their dominant water uptake mechanism. For example [21], recently showed that the equation may not be valid for clays, especially at low saturations, where adsorptive forces dominate water uptake. In the case of biofilm-treated sand, water uptake is controlled by adsorptive forces in addition to capillary forces. The capillary forces in biofilm-treated sand are not the same as the capillary forces in untreated sand because capillarity may result in water retention within the biofilm itself as well as in sand pores.

### Unconfined compression tests and SSCC of biofilm-treated sand

The unconfined compressive strength (*q*_*u*_) of cylindrical specimens was measured using a standard universal testing machine. The samples were loaded until failure, and peak stresses are reported as *q*_*u*_. The relative humidity in the laboratory fluctuated between ~10% and ~30%, which resulted in some moisture loss during the unconfined compression test. A strain rate of 0.4% was selected to ensure all specimens would fail within 10 min, and therefore the moisture loss during testing was minimal.

The SSCC of biofilm-treated sand was calculated using *q*_*u*_ data according to Refs. [30,31]:

(3a) $$q=q_{u}=\sigma_{1}$$

(3b) $$p=\frac{\sigma_{1}}{3}$$

(3c) $$p^{\prime }=q/m$$

(3d) $$\sigma^{s}=p-p^{\prime }$$

where *p’* is the mean effective stress, *p* is the mean stress, *q* is the deviatoric stress, and *m* is related to the internal friction angle, ϕ:

(4) $$m=\frac{6sin\phi }{3-sin\phi }$$

The internal friction angle is assumed to be independent of saturation and matric suction, as was shown in previous studies (e.g., Refs. [29,32]); therefore, *m* does not change with saturation. The effect of biofilms on soil mechanical behavior is assumed to only influence SSCC, rather than changing the frictional resistance between sand particles; this too, was shown in previous studies [33]. Thus, the internal friction angle of the untreated dry sand was measured to perform SSCC calculations from *q*_*u*_ data. Direct shear test was performed according to ASTM D3080. Dry sand was compacted in the shear box at a 0.85 void ratio. The test was performed at a shearing rate of 0.5 mm/min. The internal friction angle was calculated as 34°.

### Water vapor sorption isotherms and specific surface area

A fully automated vapor sorption analyzer (VSA, METER Group, Pullman, WA) operating in dynamic dewpoint isotherm (DDI) mode was used to measure the isotherm following [23]. The granules were oven-dried (40 °C) for 2 h prior to testing. Dried granules (0.6 g) were brought to 3% relative humidity (RH) followed by an adsorption cycle up to 95% RH, followed by a desorption cycle back down to 3% RH in 1% RH increments at a controlled temperature of 25 ± 0.2 °C.

## Results and discussion

### Unconfined compressive strength

**Fig. 2:**
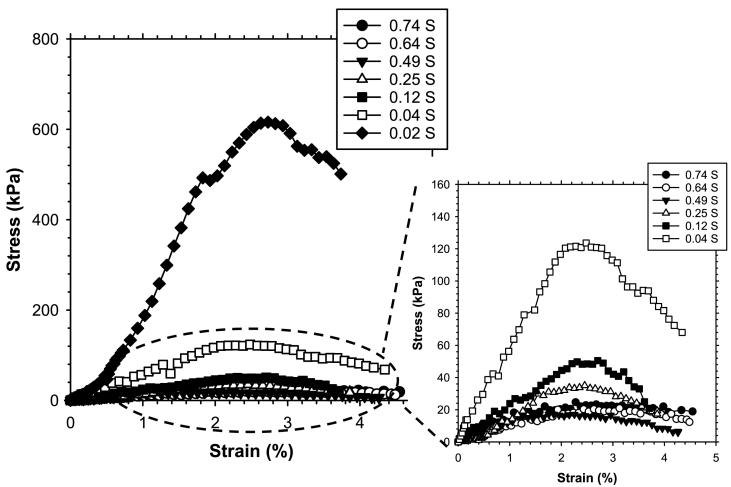
Stress-strain behavior of biofilm-enhanced sand over a wide range of saturation.

The stress-strain behavior of biofilm-treated sand was measured over a wide range of saturation (Fig. 2). While the peak strength increased with decreasing saturation, there was no considerable relationship between peak strain and saturation. All specimens reached peak strength at ~2.5% strain. Specimens with higher saturations (*S* ≥ 0.5) showed a ductile stress-strain response, whereas specimens at lower saturations showed post-peak stress. The ductile stress-strain response is attributed to a well-developed hydrogel structure between sand particles. The unconfined compressive strength decreased with saturation as shown in Fig. SI-2. All specimens failed along a vertical line as shown in Fig. SI-3, suggesting non-homogeneity of the specimens was not a concern.

### Suction stress characteristic curve of treated and untreated sand

**Fig. 3:**
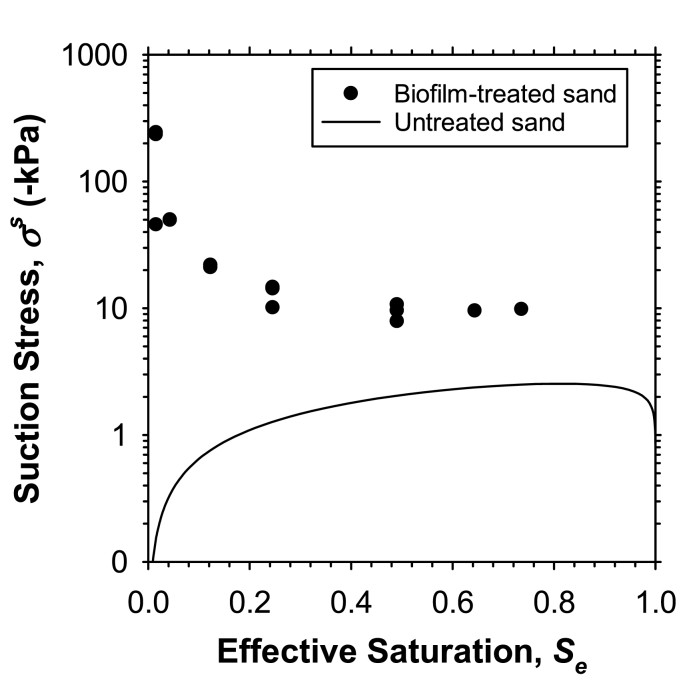
Suction stress characteristic curve (SSCC) of biofilm-enhanced sand (symbols) and untreated sand (solid line). The difference between two curves indicates the improvement in sand strength due to biofilm.

The SSCC of untreated sand followed the typical trend for sand of zero suction stress at saturations of 0 and 1 and a single peak in between (line in Fig. 3). The maximum suction stress of around 2 kPa was obtained at a saturation around 0.7 and was maintained up to a saturation of ~0.97. The SSCC of biofilm-enhanced sand (symbols in Fig. 3) showed an exponential decrease from 245 kPa to 10 kPa as the saturation increased from 0.02 to 0.5, followed by a constant suction stress of 10 kPa that was maintained up to 0.74 *S*. Air-drying time to desaturate the specimens only controls the saturation. Since biofilm growth is negligible under air-drying conditions, the air-drying time does not reflect the curing process in traditional soil improvement methods. The effect of air-drying in our case was only a reduction in saturation. Therefore, the results show the sensitivity of the strength of biofilm-enhanced soil to saturation, especially in the low-saturation range, where water uptake is controlled by adsorptive mechanisms.

The trend seen in the SSCC at low saturations is typical for a clayey soil, where adsorptive forces result in an initially high suction stress at 0 saturation that progressively decreases as sorptive sites on the clay surfaces are occupied by progressively increasing numbers of water molecules (e.g., Refs. [20,21,34]). The suction stress of untreated sand was zero at 0 saturation; in contrast, the treated sand showed a 245-kPa suction stress because of the cementation component and, more profoundly, because of the strong adsorptive interactions among water molecules, EPS, biofilm surfaces, and sand. The exponential decrease in suction stress was attributed to the formation of a gel structure in biofilms that reduces the adsorptive and capillary forces within the biofilms. The 10-kPa suction stress that was maintained with further increases in saturation was attributed to the cementation component of suction stress, which is considered to be unaffected by saturation [18].

### Interaction between biofilms and sand: mechanisms

Our homogenized biofilms include cells and EPS. In general, microorganisms attach to mineral surfaces via two mechanisms [35]: (i) long-range attachment, which is controlled by physicochemical forces (i.e., van der Waals and electrostatic interactions) and Lewis acid/base hydrophobic interactions (e.g. Ref. [36]), and (ii) short-range attachment, which includes hydrogen bonding and dipole-dipole interactions (e.g., Ref. [37]). The initial attachment is followed by the multiplication of bacterial cells on the surfaces if the conditions are favorable for biofilm growth (i.e., nutrient availability, hydrodynamics, pH). During biofilm growth, the bacterial cells produce EPS which is comprised of proteins, polysaccharides, nucleic acids, and lipids [38]. EPS, referred to as the “glue of cells” by biofilm researchers, keep microbial communities together and protect the cells. EPS also attach to mineral surfaces through steric interactions [38]. In our system, we introduce existing EPS to the sand samples instead of generating EPS by growing cells.

In geotechnical engineering applications, the terms “biofilm” and “biopolymer” are used interchangeably in some references; however, here we specify the difference that “biofilm” includes both bacteria and EPS that are produced by the bacteria, while biopolymer does not include bacteria [38]. This distinction is important because it means biofilm can keep growing in the soil if there is sufficient growth medium present, whereas the biopolymer concentration does not change after the biopolymer is mixed with the soil. The resilience of bacteria is supported by EPS, which may be critical for long-term soil improvement [39]. In our manuscript, we use the term biofilm to refer to bacteria present together with EPS.

The level of interaction between biofilms and sand surfaces is controlled by saturation. Biofilm or biopolymer treatments of sand are typically done at high saturations, where the water phase is continuous (e.g., Refs. [7,14]). In a saturated medium, microorganisms are attached to non-charged sand surfaces by electrostatic interactions. Even though sand surfaces are practically neutral, electrical charges in water stimulate the adsorption of microorganisms by sand surfaces. The adsorbed microorganisms produce EPS that act as a polymer or biopolymer, which results in an increase in particle contact area. The process, referred to as biocementation, improves soil properties. In addition, biopolymer or EPS fibers can form a connected network within the pore space between sand particles and contribute to improved strength (e.g., Refs. [7,8]). Additionally, adsorption and capillary condensation results in an increase in soil strength in unsaturated conditions (e.g., Ref. [21]). The adsorption and capillary condensation by the biofilms contribute to the suction stress of the biofilm-enhanced sand (Fig. 3).

### Water vapor sorption isotherms

**Fig. 4:**
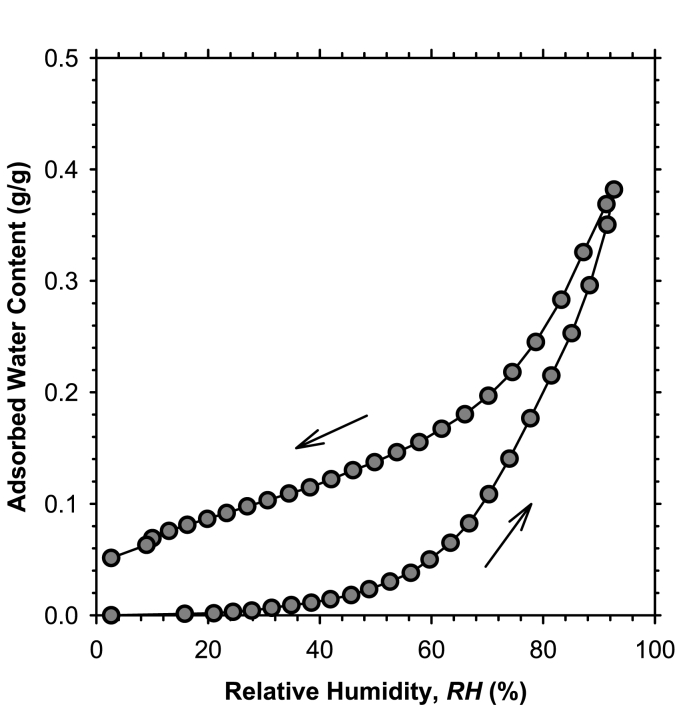
Water vapor sorption isotherm of granules.

The water vapor sorption isotherm (Fig. 4) of the biofilm granule was measured to evaluate the degree of adsorptive forces. The adsorption and desorption curves show two distinct slopes, which represent the rate of sorption, and a single inflection point, which represents the transition between water uptake mechanisms [20]. A milder slope up to ~60% RH for adsorption and ~70% RH for desorption is followed by a steeper slope up to 95% RH. As RH increases, there are two mechanisms that increase the slope of the isotherm: (i) onset of capillary condensation in mesopores and (ii) onset of formation of the hydrogel structure. The transition point is at ~60% RH for the adsorption curve and at ~70% RH for the desorption curve.

Hysteretic behavior is typical for water sorption by porous materials, and is primarily due to capillary condensation in mesopores. However, the loop is typically closed at 3% RH (e.g., Refs. [23,40,41]). Fig. 4 shows that the sorption loop was not closed for the biofilm granules, indicating that irreversible changes happened to the biofilm structure at the end of a full sorption cycle. The nonzero water content at 3% RH on the desorption curve indicates there was entrapped water in the biofilm structure that could not be removed with a decrease in RH. The specific surface area of the granules were calculated from the desorption isotherm according to Ref. [23] as 280 m^2^/g, which indicates high surface activity, and therefore strong adsorptive forces between biofilm granules and water molecules.

The SSCC of biofilm-enhanced sand (Fig. 3) shows an inflection point at ~0.3 saturation, which corresponds to a water content of 0.1 g/g. Suction stress starts to level down at ~10 kPa at this water content, which corresponds to a RH of ~35% (Fig. 4). This indicates that strong adsorptive forces among water, biofilm surfaces, and EPS contribute to an order of magnitude greater suction stress at low saturations, where the dominant water uptake mechanism is adsorption.

## Conclusions

The biofilm granules used in this study were shown to contribute to suction stress, and therefore to soil strength, in a wide range of saturations. Suction stress of biofilm-enhanced sand decreased exponentially with increasing saturation up to ~0.5 S and with further increase in saturation was maintained at ~10 kPa, which is an order of magnitude greater than the suction stress of untreated sand. The initial reduction at low saturations up to the inflection point (S < 0.3) resulted in an order of magnitude difference between the calculated suction stress. The specific surface area of the granules was calculated from the water vapor sorption isotherm as 280 m^2^/g, which indicates high surface activity, and therefore strong adsorptive forces between biofilm granules and water molecules. The nonzero water retention in the desorption cycle at 3% RH indicates possible hydrogel formation during adsorption and irreversible changes to granule structure. Since most of the microbes in the biofilms were anaerobic, they will have limited metabolic activity in the given conditions. Therefore, the main driving force for improving the soil mechanical behavior is the EPS presented in the biofilm which forms a hydrogel when bound to water [38].

## CRediT authorship contribution statement

**Ahmad Faysal Shariq:** Investigation, Validation, Formal analysis, Writing – review & editing. **Haluk Beyenal:** Conceptualization, Resources, Writing – review & editing, Supervision. **Idil Deniz Akin:** Conceptualization, Resources, Visualization, Writing – review & editing, Supervision.

## Declaration of competing interest

The authors do not have any competing interest.
