# Supplementary material for: Biofilm addition improves sand strength over a wide range of saturations
Source: Biofilm. 2021 Jun 10;3:100050. doi: 10.1016/j.bioflm.2021.100050 (PMC8260866; doi:10.1016/j.bioflm.2021.100050)
Supplement: Multimedia component 1 [file mmc1.docx]

**SUPPLEMENTARY INFORMATION for**

**Biofilm addition improves sand strength over a wide range of saturations**

**Previous literature on biofilm-enhanced soil behavior**

Early studies on the effects of biofilms on soil behavior focused on hydraulic conductivity reduction to investigate biological clogging (i.e., bioclogging) mechanisms in infiltration ponds in wastewater treatment systems and field water injection systems used in petroleum reservoirs (e.g., Mitchell and Nevo 1964, Kalish et al. 1964, Shaw et al. 1985). Bioclogging has also been observed in waste containment systems, in both compacted clay liners and leachate collection systems, and more studies were conducted to determine the effect of landfill leachate on bioclogging and hydraulic conductivity (e.g., Francisca and Glastein 2010).

Laboratory-controlled studies focused on using biofilms to modify the mechanical and hydraulic behavior of soils have been very limited. The outcomes of these studies are summarized in Table 1. The engineering properties of soils that have been studied are limited to hydraulic conductivity (Shaw et al. 1985, Dennis and Turner 1998, Lozada et al. 1994) and shear wave velocity (Ta 2016). The bacteria used in these studies include *Beijerinckia indica, Micrococcus roseus, Proteus vulgaris, Shewanella oneidensis, Pseudomonas putida, Pseudomonas sp., Methanosarcina barkeri,* and *Flavobacterium johnsoniae*, and the soil types that have been used are sand and silty sand. Only three studies on the strength of biofilm-enhanced sand were found in the literature. Gyr (1998) treated sand with *Klebsiella oxytoca*, which resulted in no change in effective stress. Banagan et al. (2010) treated sand with *Flavobacterium johnsoniae* and found that shear strength, measured with the vane shear test, increased up to 87.3%. Lin et al. (2018) treated Ottawa sand with *Staphylococcus epidermidis* and found, with the triaxial test, that the treated sand in fact had 9% less shear strength than the untreated sand.

**Table 1.** Summary of the outcomes of biofilm-enhancement studies in the literature; *k* is hydraulic conductivity and *k_r_* is the hydraulic conductivity ratio of the biofilm treated and untreated specimens.

| Bacteria | Soil Type  ([k] = cm/s) | Test | Engineering Property | Bioenhancement Effectiveness | Reference |
| --- | --- | --- | --- | --- | --- |
| *Beijerinckia indica* | Silty sand  (k_u_ = 2x10^-5^) | Flexible-wall | k for tap water | $k_{r}=2.5x{10}^{-3}$ | Dennis and Turner (1998) |
| *Klebsiella oxytoca* | Ottowa sand  (k_u_= 1.2x10^-3^) | Falling head  Triaxial | k  q and p’ | $k_{r}=7.3*{10}^{-2}$  No change | Gyr (1998) |
| *Micrococcus roseus (d)*  *Proteus vulgaris (d)*  *Micrococcus flavus (d)* | Berea sand stone  (k_u_ = 3x10^-4^) | Falling head | k | $k_{r}=3.3x{10}^{-1}$  $k_{r}=3x{10}^{-1}$  $k_{r}=5x{10}^{-2}$ | Kalish et al. (1964) |
| *Pseudomonas sp. (l)*  *Pseudomonas sp. (d)* | Sintered glass bead model cores  (k_u_ = 6x10^-3^) | Falling head | k | $k_{r}=3.3x{10}^{-3}$  $k_{r}=6.6x{10}^{-1}$ | Shaw et al. (1985) |
| *Shewanella oneidensis*  *Pseudomonas putida* | Ottawa Sand  (k_u_ = 1x10^-4^) | Falling head | k | $k_{r}=2.4x{10}^{-1}$  $k_{r}=1.8x{10}^{-1}$ | Ta (2016) |
| *Methanosarcina barkeri* | Quartz sand | Constant head | k | 97% and 75% decrease | Lozada et al. (1994) |
| *Flavobacterium johnsoniae* | Ottawa sand | Vane shear test | shear strength | up to 87.3% increase | Banagan et al. (2010) |
| *Staphylococcus epidermidis* | Ottawa sand 50/70 | Triaxial | Shear strength | 9% decrease | Lin et al. (2018) |

**Drying time**

Fig. SI-1 shows the change in saturation with increasing air-drying time. Saturation decreased exponentially with air-drying time.

**Figure SI-1.** Specimen conditioning: change in saturation with increasing air-drying time.

**Unconfined compressive strength**

The value of the unconfined compressive strength, *q_u_* decreased exponentially from 620 kPa to 25 kPa as saturation increased from 0.02 to 0.5 and was maintained around 25 kPa as saturation increased further (Fig. SI-2). The most profound reduction occurred when saturation increased from 0.02 to 0.04, and *q_u_* decreased by 4.9 times. An increase in saturation to 0.1 decreased *q_u_* by half.

***
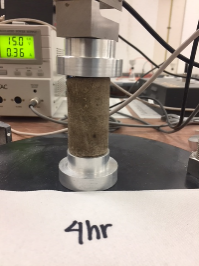
***

**Figure SI-2.** UCS of biofilm-enhanced sand over a wide range of saturation. A specimen undergoing the unconfined compressive strength test is shown in the inset.

**Failure envelopes**

The specimens failed along the vertical diameter and the failure envelope of the specimens did not indicate non-homogeneity. Fig. SI-3 shows representative images of failed specimens for specimens dried for 8h, 72 h, 96 h, and 120 h.


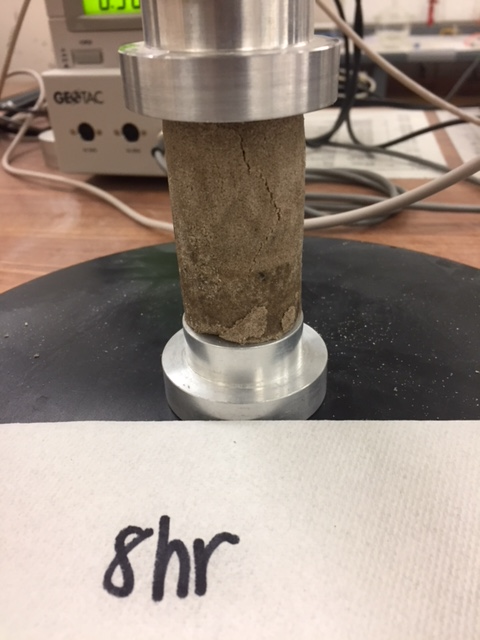

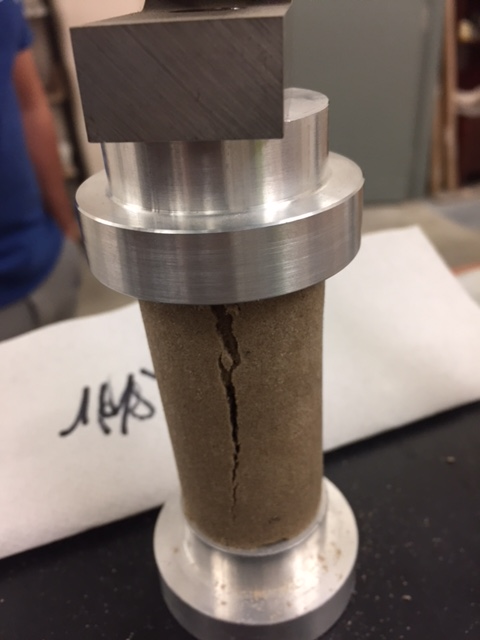

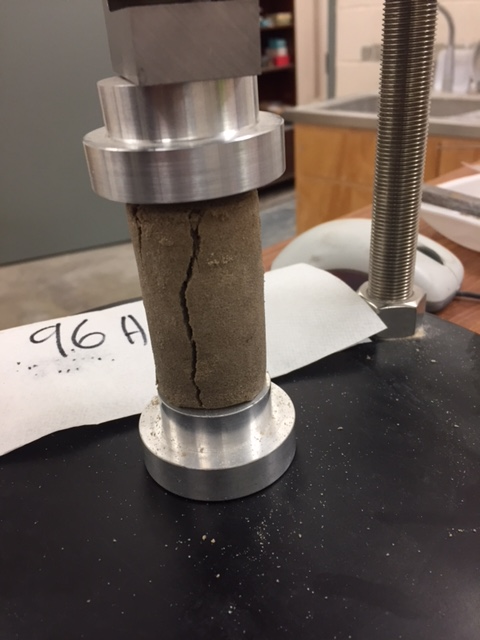

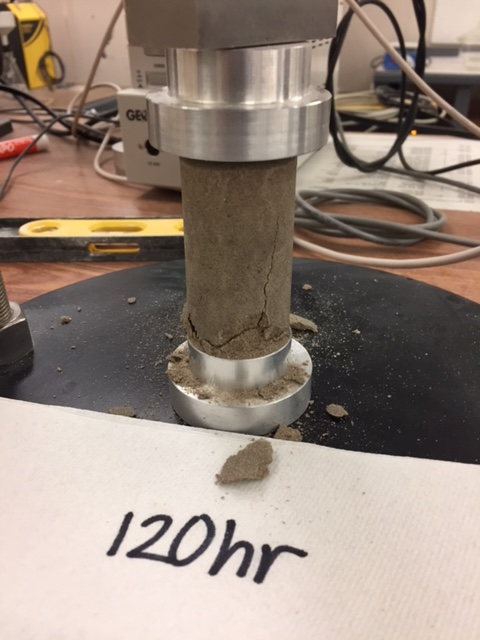


**Figure SI-3.** Representative images of failed specimens for specimens dried for 8h, 72 h, 96 h, and 120 h.

**References**

Banagan, B. L., Wertheim, B. M., Roth, M. J. S., and Caslake, L. F. 2010. “Microbial strengthening of loose sand.” *Letters in applied microbiology*, 51(2), 138-142.

Dennis, M. L., and Turner, J. P. 1998. “Hydraulic conductivity of compacted soil treated with biofilm.” *Journal of Geotechnical and Geoenvironmental Engineering*, 124(2), 120-127

Francisca, F. M., and Glatstein, D. A., 2010. “Long term hydraulic conductivity of compacted soils permeated with landfill leachate,” *Applied Clay Science*, 49, pp. 187 - 193.

Gyr., P. 1998. The mechanical properties of biofilm populated sand. MS Thesis, Montana State University, Bozeman, Montana.

Kalish, P. J., Stewart, J. A., Rogers, W. F., & Bennett, E. O. 1964. “The effect of bacteria on sandstone permeability,” *Journal of petroleum technology*, *16*(07): 805-814.

Lin, H., Suleiman, M.T., and Brown, D.G. 2018. “Behavior of biofilm-treated sand.” *IFCEE*

*2018 Technical Papers*, pp. 1-11.

Lozada, D. S., Vandevivere, P., Baveye, P., and Zinder, S., 1994. “Decrease of the hydraulic conductivity of sand columns by *Methanosarcina barkteri*,” *World Journal of Microbiology & Biotechnology,* 10, pp. 325 - 222.

Mitchell, R., and Nevo, Z. 1964. “Effect of bacterial polysaccharide accumulation on infiltration of water through sand.” *Appl. Environ. Microbiol.*, 12(3), 219-223.

Shaw, R. J., Bramhill, B., Wardlaw, N. C., and Costerton, J. W., 1985. “Bacterial fouling in a model core system,” Applied and Environmental Microbiology, p. 693 - 701.

Ta, H.X. 2016. “Microbial biofilm in porous sediments: effects on soil behavior.” PhD Dissertation, Washington State University, Pullman, Washington.
